# A preliminary evaluation of quercetin-mediated osteogenic gene expression in lipopolysaccharide-treated human periodontal ligament cells: an in vitro study

**DOI:** 10.1038/s41405-026-00434-z

**Published:** 2026-04-27

**Authors:** Srishta Radhakrishnan, Prem Blaisie Rajula M, P. L. Ravi Shankar, Merita S, V. Kalaivani, Rahila C

**Affiliations:** 1 Department of Periodontology, SRM Kattankulathur Dental College and Hospital, Faculty of Medicine and Health Sciences, SRM Institute of Science and Technology, Kattankulathur, Chengalpattu, Tamil Nadu India; 2 Department of Public Health Dentistry, Vivekananda Dental College for Women, Dr. MGR Medical University, Chengalpattu, Tamil Nadu India

**Keywords:** Periodontitis, Periodontics, Periodontitis

## Abstract

**Aim:**

This study aimed to evaluate the effect of quercetin on osteogenic gene expression in lipopolysaccharide (LPS) stimulated human periodontal ligament cells (hPDLCs) under in vitro conditions.

**Materials and methods:**

hPDLCs were cultured with LPS for 24 hours to simulate an inflammatory microenvironment. Following this pre-stimulation, cells were treated with quercetin at concentrations of 2.5 µM, 5 µM, and 10 µM for up to 14 days. No further exposure to LPS was performed during the subsequent culture period. The mRNA expression levels of osteogenic markers, osteopontin (OPN) and osteocalcin (OCN), were assessed on day 14 using Quantitative Real-Time PCR (RT-qPCR). Statistical analysis was performed using Welch’s one-way ANOVA with Holm-adjusted post hoc comparisons.

**Results:**

LPS stimulation significantly suppressed the expression of both OPN and OCN compared with the control group. However, quercetin treatment restored and dose-dependently increased the expression of these markers, with the greatest effect observed at 10 µM. At this concentration, OPN and OCN expression levels reached 5.80 ± 0.26 and 6.62 ± 0.30, respectively, relative to the control. A consistent dose-dependent upregulation was observed for both markers, indicating restoration of osteogenic gene expression.

**Conclusion:**

Quercetin modulates the expression of osteogenic markers, including OPN and OCN, in LPS-stimulated hPDLCs under in vitro conditions. These findings suggest a potential modulatory role for quercetin in influencing osteogenic gene expression. However, additional in vitro functional assays and in vivo studies are necessary to establish its role in periodontal regeneration.

## Introduction

Periodontal disease is a chronic inflammatory condition encompassing gingivitis and periodontitis, that causes progressive destruction of the periodontium, ultimately resulting in tooth loss if left untreated [1]. It is particularly prevalent among the elderly population and is not uncommon in younger individuals [2]. The oral cavity contains around 700 species of bacteria, thus forming a unique and complex microbial environment with diverse ecosystem, many of which play a vital role in the onset and advancement of periodontitis [3].

Human periodontal ligament cells (hPDLCs) are primary cells isolated from periodontal ligament tissue and represent a heterogeneous population predominantly composed of fibroblastic cells, along with smaller proportions of osteoblast-like, cementoblast-like, and stem-like cells. Within this mixed population, periodontal ligament stem cells (PDLSCs) constitute a distinct subpopulation that exhibits mesenchymal stem cell–like properties, including osteogenic differentiation potential and regenerative capacity [4]. Due to their multipotential capacity to differentiate into various types of cells, PDLSCs can modulate immune responses, thereby enabling effective and sustained periodontal repair. However, in the absence of definitive stem cell characterisation, primary periodontal ligament cultures are more appropriately described as hPDLCs rather than PDLSCs. Therefore, hPDLCs serve as a biologically relevant in vitro model for investigating periodontal regeneration and inflammatory responses [5].

Under inflammatory periodontal conditions, hPDLCs are exposed to bacterial components such as lipopolysaccharide (LPS), a potent endotoxin present in the outer membrane of Gram-negative bacteria [6]. LPS is a significant microbiological contributor to periodontal disease as it forms the initial interface between bacterial pathogens and the human immune system. LPS induces inflammatory responses that degrade bone and connective tissue. As the disease progresses, LPS elevates the concentrations of pro-inflammatory cytokines, including interleukins and C-reactive proteins [7]. It results in gingival bleeding, recession, and in extreme cases, mobility and tooth loss [8]. Among gram-negative bacteria, *Porphyromonas gingivalis* is regarded as the keystone pathogen in the pathophysiology of periodontitis. The LPS of *P. gingivalis* is particularly effective in sustaining chronic inflammation by activating Toll-like receptors (TLRs), notably TLR4, which in turn initiates downstream signalling cascades involving transcription factors like nuclear factor-kappa B (NF-κB), thus resulting in the synthesis of diverse inflammatory mediators [9].

Recognising the constraints of traditional treatment approaches, natural compounds with anti-inflammatory and regenerative properties have gained significant interest. Quercetin (Q), a plant-derived polyphenolic flavonoid, is abundantly present in fruits (e.g., apples, berries, grapes), vegetables (e.g., onions, broccoli, cabbage), and other plant parts like seeds and leaves [10, 11]. Quercetin is known for its broad-spectrum pharmacological activities, including anti-allergic, anti-inflammatory, anti-tumour, antiviral, and cardiovascular protective effects [12]. It has been approved by the U.S. FDA as a component in antioxidant and anti-allergic formulations [13].

Mechanistically, quercetin exerts its anti-inflammatory effects by inhibiting cyclooxygenase (COX) and lipoxygenase (LOX) enzymes, which are key mediators in the arachidonic acid pathway [9]. Moreover, quercetin has been demonstrated to reduce oxidative stress, diminish osteoblast apoptosis, and inhibit RANKL-induced osteoclastogenesis [14].

In the present study, the effect of quercetin on osteogenic gene expression was evaluated in an LPS-induced inflammatory environment using hPDLCs by analysing the expression of osteogenic gene markers, osteopontin (OPN) and osteocalcin (OCN).

## Materials and methods

### Chemicals and consumables

All chemicals and consumables used in this study were of analytical or cell culture grade. Quercetin (≥95% purity; Cat. No. Q4951) and lipopolysaccharide from *Porphyromonas gingivalis* (LPS; Cat. No. SMB00610) were procured from Sigma-Aldrich (USA). Dulbecco’s Modified Eagle Medium, high glucose (DMEM; Cat. No. 11965-092), fetal bovine serum (FBS; Cat. No. 10270-106), penicillin–streptomycin solution (Cat. No. 15140-122), and trypsin–EDTA (0.25%; Cat. No. 25200-056) were obtained from Gibco™, Thermo Fisher Scientific (USA). Collagenase type I (Cat. No. 17100017) and dispase II (Cat. No. 17105041) were purchased from Thermo Fisher Scientific (USA). MTT reagent (Cat. No. M5655) and dimethyl sulfoxide (DMSO; Cat. No. D4540) were obtained from Sigma-Aldrich (USA). Phosphate-buffered saline (PBS; Cat. No. TS1006), TRIzol reagent (Cat. No. 15596026), and Power SYBR™ Green Master Mix (Cat. No. 4367659) were sourced from Thermo Fisher Scientific (USA). Primers were synthesised by a commercial supplier (Eurofins/Integrated DNA Technologies). All plasticware including culture plates, centrifuge tubes, and pipette tips were procured from HiMedia Laboratories (India) and SRL (India).

### Isolation and cultivation of human periodontal ligament cells (hPDLCs)

Human periodontal ligament cells (hPDLCs) were isolated following a standardised protocol routinely employed in our laboratory for primary periodontal cell culture studies. Briefly, periodontally healthy premolars (*n* = 3), extracted for orthodontic purposes from three independent donors, were used as the biological source following informed consent and approval by the Institutional Ethics Committee (SRMIEC-ST0525-2583). Each tooth represented an individual biological replicate, and no pooling of samples was performed, ensuring preservation of donor-specific variability and adherence to reproducibility standards in primary cell research.

Immediately after extraction, periodontal ligament tissue was gently harvested from the middle third of the root surface using sterile scalpel blades under aseptic conditions. The collected tissue samples were subjected to enzymatic digestion using a combination of collagenase type I (3 mg/mL) and dispase II (4 mg/mL) in sterile phosphate-buffered saline. Digestion was carried out at 37 °C for 45–60 minutes under gentle agitation to facilitate efficient release of viable cells.

Following digestion, the cell suspension was passed through a sterile cell strainer to remove undigested debris and centrifuged at 3000 rpm for 5 minutes (as per standard mammalian cell handling SOP). The resulting cell pellet was resuspended in complete culture medium consisting of DMEM supplemented with 10% fetal bovine serum and 1% penicillin–streptomycin, and seeded into culture flasks.

Cells were maintained at 37 °C in a humidified atmosphere containing 5% CO₂, and media were changed every 2–3 days. Upon reaching 80–90% confluence, cells were passaged using trypsin–EDTA. Cells at passages 2–4 were used for all experiments to ensure phenotypic stability and optimal biological responsiveness. As stem cell enrichment or immunophenotypic characterisation (e.g., STRO-1/CD146) was not performed, the cells are consistently referred to as Human periodontal ligament cells (hPDLCs).

### Preparation of reagents

Quercetin (≥95% purity; Sigma-Aldrich, USA; Cat. No. Q4951) was dissolved in dimethyl sulfoxide (DMSO; Sigma-Aldrich; Cat. No. D4540) to prepare a stock solution and diluted in culture medium to final concentrations (2.5, 5, and 10 µM). The final DMSO concentration did not exceed 0.1% (v/v). LPS from *Porphyromonas gingivalis* (Sigma-Aldrich; Cat. No. SMB00610) was prepared according to the manufacturer’s instructions and directly diluted in complete culture medium (Hi Media, India; Cat. No. TS1006) to achieve the desired working concentration (10 µg/mL) [5]. To ensure uniform solvent exposure and eliminate vehicle-related bias, an equivalent concentration of DMSO (0.1% v/v) was added to the Control and LPS groups. All reagents were freshly prepared before use.

### Pilot cytocompatibility assessment of quercetin on hPDLCs (MTT assay)

Pilot cytocompatibility of quercetin was evaluated using the MTT assay in accordance with our laboratory’s standardised SOP for biomaterial and phytochemical screening. Human periodontal ligament cells (hPDLCs; passage 2–4) were seeded in 96-well plates at a density of 5 × 10³ cells/well and incubated for 24 hours to allow cell attachment. Cells were then treated with quercetin at 2.5 µM, 5 µM, and 10 µM [15], along with a vehicle control (0.1% DMSO) for 24 hours. Following treatment, 20 µL of MTT reagent (5 mg/mL in PBS) was added to each well and incubated for 4 hours at 37 °C. The resulting formazan crystals were dissolved in 100 µL of dimethyl sulfoxide (DMSO), and absorbance was measured at 570 nm using a microplate reader. Experiments were performed in triplicate (*n* = 3 biological replicates), each with technical duplicates, and cell viability was expressed as a percentage relative to the vehicle control (Table S1 and Fig. S1).

### Experimental design and treatment groups

Cells were seeded in appropriate culture plates and allowed to reach approximately 70–80% confluence prior to treatment. Lipopolysaccharide (LPS) was used to induce an inflammatory microenvironment that mimics periodontitis. Cells were first stimulated with LPS for 24 hours to establish inflammation, after which quercetin treatment was initiated and maintained continuously throughout the subsequent osteogenic differentiation period, up to Day 14. Experimental groups were designed as follows: Group I (Vehicle Control): hPDLCs with 0.1% DMSO, Group II (LPS): LPS-stimulated hPDLCs, Group III (LPS + Quercetin at 2.5 µM): LPS-stimulated hPDLCs treated with quercetin (2.5 µM), Group IV (LPS + Quercetin at 5 µM): LPS-stimulated hPDLCs treated with quercetin (5 µM), Group V (LPS + Quercetin at 10 µM): LPS-stimulated hPDLCs treated with quercetin (10 µM).

### Induction of inflammation and osteogenic differentiation

To induce inflammation, hPDLCs were exposed to LPS for 24 hours. Following inflammatory induction, cells were treated with quercetin as per group allocation for up to 14 days. No further exposure to LPS was performed during the subsequent 14-day culture period.

For osteogenic differentiation, cells were cultured in osteogenic induction medium consisting of basal DMEM (Gibco, Thermo Fisher Scientific; Cat. No. 11965-092) supplemented with 10% FBS (Gibco; Cat. No. 10270-106), β-glycerophosphate (10 mM; Sigma-Aldrich, Cat. No. G9422), ascorbic acid (50 µg/mL; Sigma-Aldrich, Cat. No. A4544), and dexamethasone (100 nM; Sigma-Aldrich, Cat. No. D4902). Osteogenic medium was refreshed every 2–3 days, and cells were maintained under osteogenic conditions for 14 days. All experimental groups, including the vehicle control, were cultured in the same osteogenic induction medium throughout the study period to ensure consistency. Osteogenic differentiation was evaluated by assessing the expression of osteogenic gene markers, osteopontin (OPN) and osteocalcin (OCN), at day 14 using RT-qPCR.

### RNA isolation and quantitative real-time PCR (RT-qPCR)

Total RNA was extracted from cells at Day 14 (osteogenic phase) using TRIzol reagent (Thermo Fisher Scientific, Waltham, MA, USA) according to the manufacturer’s instructions. RNA quantity and purity were assessed by UV spectrophotometry at 260/280 nm using a NanoDrop ND-1000 spectrophotometer (Thermo Fisher Scientific, Waltham, MA, USA).

Complementary DNA (cDNA) was synthesised from 1 µg of total RNA using the PrimeScript RT Reagent Kit (Perfect Real Time; Takara Bio Inc., Shiga, Japan), following the manufacturer’s protocol. Quantitative real-time PCR (RT-qPCR) was performed using KAPA SYBR® Fast Universal Master Mix (2×) (Kapa Biosystems, Sigma-Aldrich, Darmstadt, Germany) on a CFX Connect™ RT-qPCR Detection System (Bio-Rad Laboratories, Hercules, CA, USA) to evaluate the expression of osteogenic markers, osteopontin (OPN) and osteocalcin (OCN).

Primer-BLAST (NCBI) was employed for primer design, and the primer sequences for GAPDH, OCN and OPN are listed in Table 1. The primers were validated for specificity (single melt-curve peaks), efficiency (90-110%), amplicon size (80-150 bp), with no evidence of primer-dimer formation (Table S2). Cycling conditions were uniform across all targets: initial denaturation at 95 °C for 3 minutes, followed by 40 cycles of 95 °C for 3 seconds and 58 °C for 30 seconds. Data were analysed using Bio-Rad CFX Manager software (version 3.0). Relative gene expression was calculated using the 2^−ΔΔCq method, normalised to the housekeeping gene GAPDH. All reactions were performed in technical duplicates, and analysis was conducted for samples collected at Day 14 only.

**Table 1:** Primer sequences for GAPDH, OCN and OPN.

Gene	Forward Primer (5′–3′)	Reverse Primer (5′–3′)
GAPDH	AGCCACATCGCTCAGACAC	GCCCAATACGACCAAATCC
OCN	CAGCGAGGTAGTGAAGAGACC	AGAGCGACACCCTAGACCG
OPN	AGCCACAAGTTTCACAGCCACA	TCGTCATCATCATCGTCATCATCC

### Biological and technical replicates

All experiments were conducted with three independent biological replicates (*n* = 3), corresponding to independent cell preparations. For RT-qPCR analysis, each biological sample was analysed in technical duplicate, and the mean of the technical duplicates was used as a single biological replicate for statistical analysis.

### Statistical analysis

Statistical analyses were performed in R version 4.5.1 (R Foundation for Statistical Computing, Vienna, Austria) using the packages *rstatix*, *Kendall*, and *clinfun*. RT-qPCR data were normalised as ΔCt = Ct(target) − Ct(GAPDH), centred to control as ΔΔCt, and expressed as fold-change (2^−ΔΔCt) for descriptive presentation. All statistical testing was conducted on ΔCt values, which are additive and approximately variance-stable.

Group differences were evaluated using Welch one-way ANOVA, followed by Holm-adjusted Welch pairwise comparisons for pre-specified contrasts (LPS vs Quercetin 2.5, 5, and 10 µM; two-tailed α = 0.05). Dose–response relationships were assessed using a directional Kendall’s τ trend test on ΔCt values, testing for decreasing ΔCt with increasing quercetin concentration. Individual biological replicate (*n* = 3 per group) values are shown in the figures, and all data are presented as mean ± standard deviation (SD).

### Ethics approval

Ethical approval for this study was obtained from the Institutional Ethics Committee of SRM Kattankulathur Dental College and Hospital (Approval No. SRMIEC-ST0525-2583). Written informed consent was obtained from all participants for the use of their extracted teeth for research purposes. Animal subjects: All authors have confirmed that this study did not involve animal subjects or tissue. Payment/services info: All authors have declared that no financial support was received from any organisation for the submitted work. Other relationships: All authors have declared that there are no other relationships or activities that could appear to have influenced the submitted work.

## Results

**Fig. 1:**
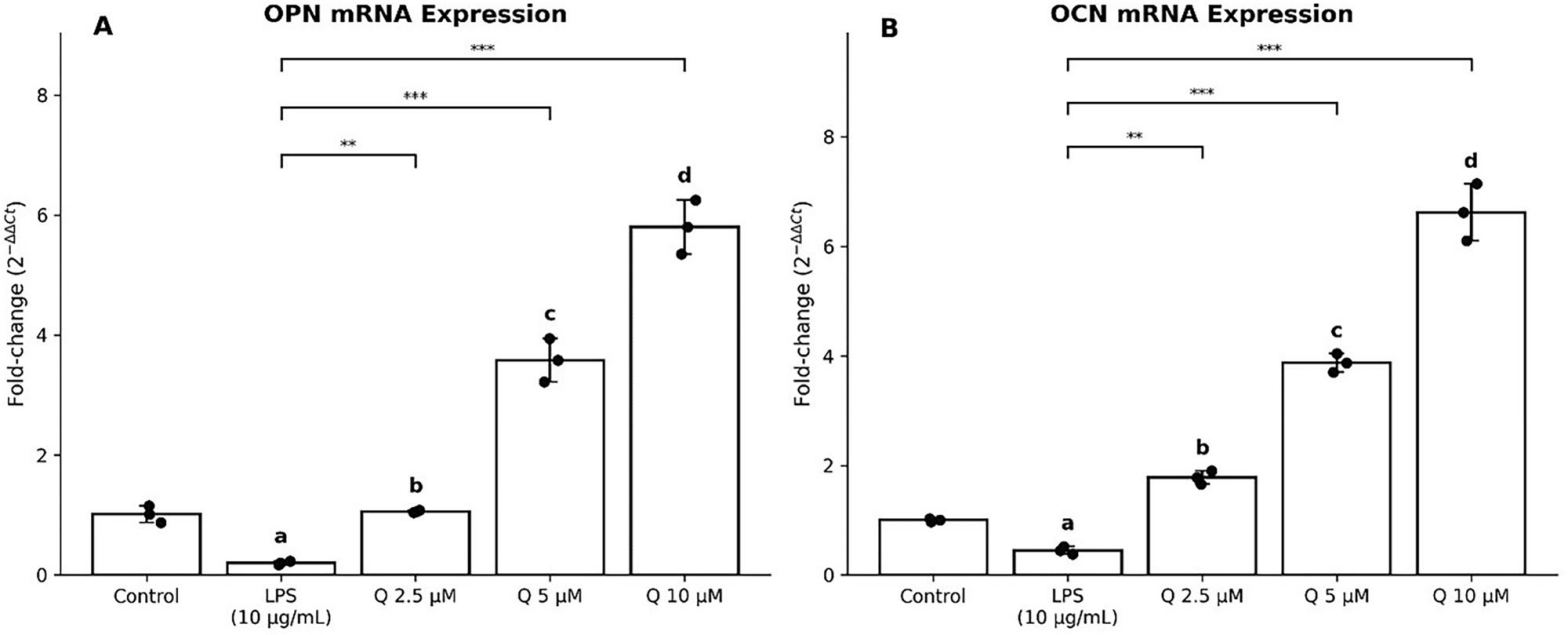
Effect of Quercetin (Q) on osteogenic gene expression in LPS-treated hPDLCs. **A** OPN and (**B**) OCN mRNA expression shown as fold-change (2^−ΔΔCt) relative to control. LPS reduced expression, while quercetin (Q; 2.5–10 µM) increased expression in a dose-dependent manner. Bars represent mean ± SD, with individual biological replicates (*n* = 3) shown as dots. Groups with different letters indicate *p* < 0.01; brackets denote significance versus LPS (**p < 0.01, ***p < 0.001).

**Fig. 2:**
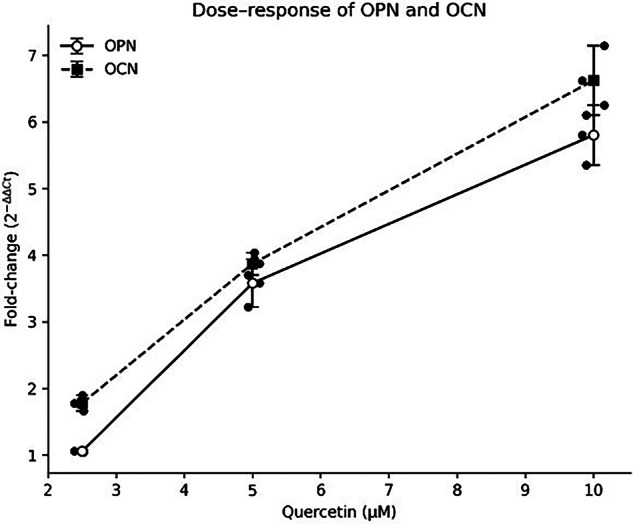
Dose–response relationship of Quercetin (Q) on OPN and OCN expression in LPS-treated hPDLCs. Mean fold-change (2^−ΔΔCt) of OPN and OCN across increasing Q concentrations (2.5–10 µM). Both markers show a dose-dependent increase. Data are presented as mean ± SD, with individual biological replicates (*n* = 3) shown as dots.

Table 2 and Fig. 1 present the relative mRNA expression of OPN and OCN. LPS reduced expression compared with control. Quercetin treatment showed a clear dose-related increase in both markers, with higher expression observed at 5 and 10 µM. While statistical testing indicated group differences, interpretation is primarily based on the consistent dose–response pattern (Fig. 2) observed across biological replicates.

**Table 2:** Relative mRNA expression of osteopontin (OPN) and osteocalcin (OCN) following quercetin treatment in LPS-treated cells.

Gene Marker	Control	LPS (10 µg/mL)	LPS + Quercetin (2.5 µM)	LPS + Quercetin (5 µM)	LPS + Quercetin (10 µM)
OPN	1.01 ± 0.14	0.20 ± 0.03^a^	1.06 ± 0.02^b^	3.58 ± 0.36^c^	5.80 ± 0.45 ^d^
OCN	1.00 ± 0.03	0.45 ± 0.07^a^	1.78 ± 0.12^b^	3.87 ± 0.17^c^	6.62 ± 0.52 ^d^

Overall group differences were evaluated using Welch one-way ANOVA on ΔCt values (OPN: F(4,10) = 461.84, *p* = 2.70 × 10⁻¹¹; OCN: F(4,10) = 501.68, *p* = 1.79 × 10⁻¹¹). Superscripts (a–d) indicate significant differences compared with the LPS group (Holm-adjusted p < 0.01). Dose–response relationships were assessed using directional Kendall’s τ (OPN: τ = −0.889, p = 0.001; OCN: τ = −0.867, *p* = 0.001). Given the small sample size (*n* = 3), results are interpreted with emphasis on consistency and magnitude of biological response.

## Discussion

Periodontal homeostasis is maintained through a tightly regulated balance between bone formation and bone resorption, a process known as coupling. In periodontitis, this balance is disrupted by chronic inflammation, leading to osteoclast-mediated bone resorption and progressive periodontal tissue destruction [16]. Persistent inflammation precedes and drives alveolar bone resorption by inducing pro-inflammatory cytokines that upregulate RANKL expression, thereby promoting osteoclast differentiation and activity while disrupting the physiological coupling between osteoblastic and osteoclastic activity [17]. The primary goal in management of periodontal disease is to halt the progression of the disease and to restore the lost periodontal tissues [18]. Non-surgical management, including scaling and root planing may be effective in mild to moderate cases but is often insufficient for deep periodontal pockets with infra-bony defects, or furcation involvements [19]. In such cases, surgical procedures, including resective and regenerative osseous surgery, combined with ongoing periodontal maintenance therapy, become necessary [20]. Although these methods assist in preventing disease progression, their capacity to regenerate the lost periodontium remains restricted [21]. These limitations emphasise the need for novel therapeutic strategies that can effectively promote tissue regeneration in the complex inflammatory microenvironment characteristic of periodontitis [22].

Human periodontal ligament cells (hPDLCs) represent a heterogeneous population, within which periodontal ligament stem cells (PDLSCs) constitute a distinct progenitor subpopulation [4]. These cells serve as a biologically relevant in vitro model for studying periodontal regeneration and inflammation. Quercetin, a naturally occurring flavonoid, has been reported to exhibit anti-inflammatory and osteogenesis-related effects in PDL cells, making it of interest in periodontal research [23, 24]. In the present study, the effect of quercetin was evaluated by analysing the expression of two osteogenic markers, osteopontin (OPN) and osteocalcin (OCN), under LPS-induced inflammatory conditions. OPN and OCN are major non-collagenous proteins associated with bone matrix formation [25]. OPN is a multifunctional signalling glycoprotein, majorly produced by immune cells, osteoblasts, osteoclasts, endothelial as well as epithelial cells. OCN, also known as the Gla-protein is the most abundant non-mineralised bone protein with high affinity to Calcium. It is secreted by osteoblasts, odontoblasts and chondrocytes [26]. OPN is generally considered an early marker of osteogenic differentiation, while OCN is a late marker associated with bone matrix deposition and mineralisation [27].

The findings of this study demonstrated that LPS stimulation was associated with reduced expression of OPN and OCN compared with the control group, indicating suppression of osteogenic marker expression under inflammatory conditions. Quercetin treatment increased OPN and OCN levels in a dose-dependent manner, reaching 1.06 ± 0.01 at 2.5 µM, 3.58 ± 0.21 at 5 µM and 5.80 ± 0.26 at 10 µM and OCN levels reaching 1.78 ± 0.07 at 2.5 µM, 3.87 ± 0.10 at 5 µM and 6.62 ± 0.30 at 10 µM. These observations suggest that quercetin influences the expression of selected osteogenic genes in LPS-stimulated hPDLCs. These findings are consistent with those of Gomez-Florit et al. who demonstrated that quercetin at a concentration of 200 µM enhanced mineralisation and alkaline phosphatase activity in human mesenchymal stem cells while attenuating inflammatory responses in gingival fibroblasts under IL-1β stimulation [28]. Similarly, the study by Wei et al. reported that quercetin at 5 µM protects hPDLCs from oxidative stress-induced senescence and preserves osteogenic potential via upregulation of ALP, Runx2, and OCN by enhancing the expression of antioxidant enzyme-related genes, including HO-1, NQO-1, GPx3 and CAT and elevated Superoxide dismutase (SOD) activity [29].

Based on the present study, quercetin upregulated the osteogenic gene expression markers in a dose-dependent manner, with 10 µM showing the highest effect. These observations suggest that quercetin may modulate osteogenic gene expression in human periodontal ligament cells (hPDLCs) under inflammatory conditions, thereby indicating its potential relevance in periodontal regeneration within an inflammatory microenvironment. However, the study has certain limitations, including the evaluation of a limited concentration range and its reliance on mRNA expression of only two osteogenic markers (OPN and OCN) without protein-level validation or functional assays such as ALP activity or mineralisation analysis. Inflammatory activity was not directly assessed, as no cytokine or signalling markers were evaluated. The study was conducted at a single time point using a 24-hour LPS pre-stimulation model, which may not fully represent sustained inflammatory conditions. Additionally, the use of heterogeneous hPDLCs, along with a limited sample size, may affect reproducibility. Nevertheless, this approach reflects the inherent heterogeneity of primary periodontal ligament cultures commonly employed in in vitro periodontal research [30, 31].

Future investigations should incorporate comprehensive stem cell characterisation, including surface marker profiling and functional differentiation assays, to further substantiate these findings. Moreover, long-term studies are warranted to assess the sustained effects of quercetin on periodontal ligament cell osteogenic behaviour. The inclusion of additional osteogenic markers, such as Runx2, COL1, ALP, and BMPs, would provide a more detailed understanding of the molecular mechanisms underlying the effects of quercetin on periodontal ligament cell behaviour.

## Conclusion

In conclusion, the present study demonstrates that quercetin reverses LPS-induced suppression of osteogenic gene marker expression in human periodontal ligament cells. These findings indicate a restoration of osteogenic gene expression under inflammatory conditions. Therefore, quercetin may be considered a potential biomodulatory agent capable of influencing osteogenic marker expression within an inflammatory microenvironment. However, further in vitro functional assays and in vivo studies are required to validate its role in periodontal regeneration.

## Supplementary information

Supplementary File

## Data Availability

The datasets of the study are available from the corresponding author on reasonable request.
