# Supplementary material for: A preliminary evaluation of quercetin-mediated osteogenic gene expression in lipopolysaccharide-treated human periodontal ligament cells: an in vitro study
Source: BDJ Open. 2026 Apr 27;12:42. doi: 10.1038/s41405-026-00434-z (PMC13121475; doi:10.1038/s41405-026-00434-z)
Supplement: Supplementary file 1 — Supplementary File [file 41405_2026_434_MOESM1_ESM.docx]

Technical duplicate consistency demonstrated minimal variation (SD < 0.1 Ct), indicating high reproducibility of RT-qPCR measurements at Day 14. Relative optical density (OD) values and percentage viability across experimental groups are presented below.

| Parameter | Control | Q 2.5 µM | Q 5 µM | Q 10 µM |
| --- | --- | --- | --- | --- |
| OD (Replicate 1) | 0.842 | 0.841 | 0.822 | 0.785 |
| OD (Replicate 2) | 0.856 | 0.835 | 0.829 | 0.789 |
| OD (Replicate 3) | 0.851 | 0.838 | 0.839 | 0.801 |
|  |  |  |  |  |
| % Viability (Replicate 1) | 100 | 99.88 | 97.62 | 93.23 |
| % Viability (Replicate 2) | 100 | 97.55 | 96.85 | 92.17 |
| % Viability (Replicate 3) | 100 | 98.47 | 98.59 | 94.12 |
|  |  |  |  |  |
| Mean (%) | 100 | 98.63 | 97.69 | 93.18 |
| SD | 0.00 | 0.96 | 0.71 | 0.80 |
| p-value | – | 0.091 | 0.023* | 0.004** |

**Table S1. Technical duplicate consistency and relative optical density (OD) measurements at Day 14**

**Footnotes:** OD: Optical Density; Values expressed as mean ± SD; *p < 0.05, **p < 0.01

| Gene | Primer Sequence (5’–3’) | Amplicon Size (bp) | Annealing Temp (°C) | PCR Efficiency (%) | R² (Standard Curve) | Melt Curve Peak (°C) |
| --- | --- | --- | --- | --- | --- | --- |
| GAPDH | AGCCACATCGCTCAGACAC  GCCCAATACGACCAAATCC | 120 | 58 | 99.2 | 0.998 | 83.5 |
| OCN | CAGCGAGGTAGTGAAGAGACC  AGAGCGACACCCTAGACCG | 132 | 58 | 98.6 | 0.997 | 84.2 |
| OPN | AGCCACAAGTTTCACAGCCACA  TCGTCATCATCATCGTCATCATCC | 145 | 58 | 97.9 | 0.996 | 85.1 |

**Table S2. Primer Validation, Amplification Conditions, and Melt Curve Analysis (RT-qPCR)**


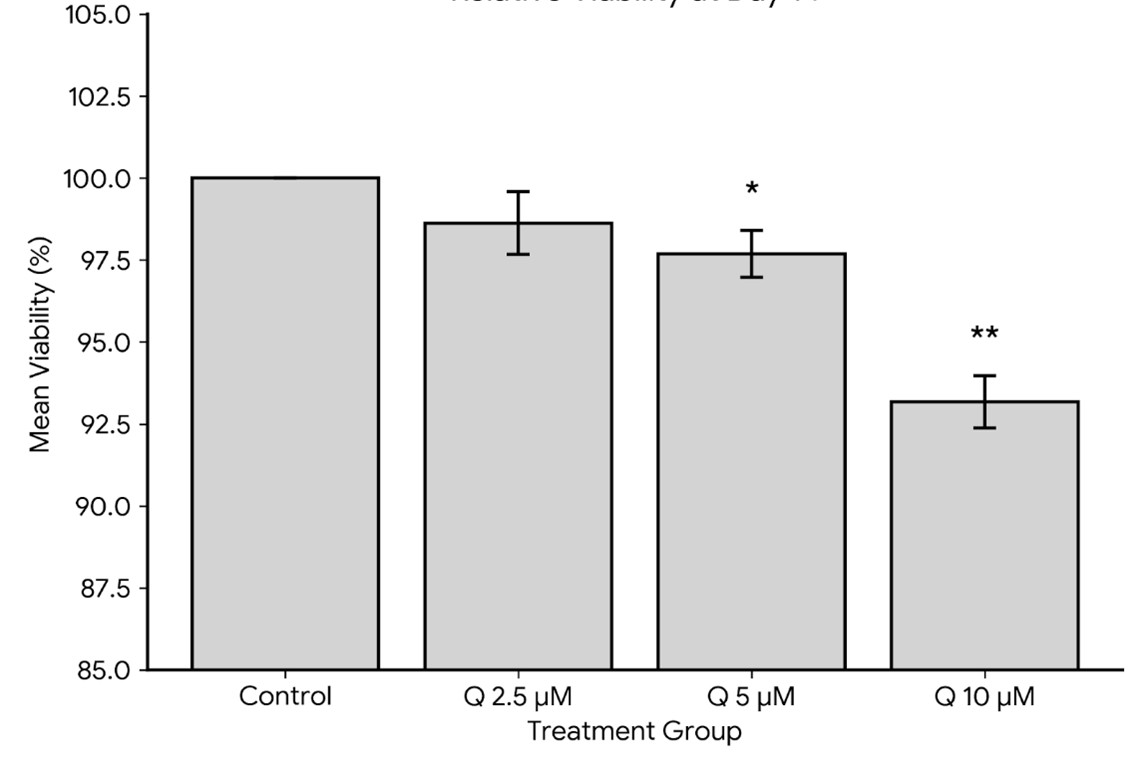


**Figure S1. Preliminary cytocompatibility assessment using MTT assay**
